# IP3R-dependent mitochondrial dysfunction mediates C5b-9-induced ferroptosis in trichloroethylene-caused immune kidney injury

**DOI:** 10.3389/fimmu.2023.1106693

**Published:** 2023-06-13

**Authors:** Zhibing Liu, Jinru Ma, Xulei Zuo, Xuesong Zhang, Haibo Xie, Feng Wang, Changhao Wu, Jiaxiang Zhang, Qixing Zhu

**Affiliations:** ^1^ Department of Dermatology, First Affiliated Hospital of Anhui Medical University, Hefei, Anhui, China; ^2^ Key Laboratory of Dermatology (Anhui Medical University), Ministry of Education, Hefei, Anhui, China; ^3^ Department of Occupational Health and Environmental Health, School of Public Health, Anhui Medical University, Hefei, Anhui, China; ^4^ Department of Nephropathy, First Affiliated Hospital of Anhui Medical University, Hefei, Anhui, China; ^5^ Department of Dermatology, Second Hospital of Anhui Medical University, Hefei, Anhui, China; ^6^ School of Biosciences and Medicine, Faculty of Health and Medical Sciences, University of Surrey, Guildford, United Kingdom

**Keywords:** occupational medicamentose-like dermatitis due to trichloroethylene, C5b-9, IP3R, mitochondrial dysfunction, ferroptosis

## Abstract

Patients with occupational medicamentose-like dermatitis due to trichloroethylene often suffer from immune kidney injury. Our previous study reveals that C5b-9-dependent cytosolic Ca^2+^ overload-induced ferroptosis is involved in trichloroethylene sensitized kidney injury. However, how C5b-9 causes cytosolic Ca^2+^ rise and the specific mechanism whereby overloaded Ca^2+^ induces ferroptosis remain unknown. The purpose of our study was to explore the role of IP3R-dependent mitochondrial dysfunction in C5b-9 mediated ferroptosis in trichloroethylene sensitized kidney. Our results showed that IP3R was activated, and mitochondrial membrane potential was decreased in the renal epithelial cells of trichloroethylene-sensitized mice, and these changes were antagonized by CD59, a C5b-9 inhibitory protein. Moreover, this phenomenon was reproduced in a C5b-9-attacked HK-2 cell model. Further investigation showed that RNA interference with *IP3R* not only alleviated C5b-9-induced cytosolic Ca^2+^ overload and mitochondrial membrane potential loss but also attenuated C5b-9-induced ferroptosis in HK-2 cells. Mechanistically, IP3R-dependent cytosolic Ca^2+^ overload activated the mitochondrial permeability transition pore, resulting in the loss of mitochondrial membrane potential and ferroptosis of HK-2 cells. Finally, cyclosporin A, a mitochondrial permeability transition pore inhibitor, not only ameliorated IP3R-dependent mitochondrial dysfunction but also blocked C5b-9-induced ferroptosis. Taken together, these results suggest that IP3R-dependent mitochondrial dysfunction plays an important role in trichloroethylene sensitized renal tubular ferroptosis.

## 1 Introduction

Trichloroethylene (TCE), a chlorinated organic compound, is a ubiquitous environmental toxicant that contaminates air, water and soil around the world (1). Despite the restrictions on TCE have reduced its use in some countries, the production of TCE remains at steady global growth, especially in the developing countries. Epidemiological investigations and animal research have revealed that exposure to TCE causes various diseases, such as nervous system, reproductive system, immune system, liver and kidney injury (2–4). In addition, some workers develop a systemic disease characterized by extensive mucocutaneous hypersensitivity reactions with multiorgan failure after occupational exposure to TCE, known as occupational medicamentose-like dermatitis due to TCE (OMDT) or TCE hypersensitivity syndrome (THS). Although the prevalence of OMDT in the occupational population exposed to TCE was reportedly in the range of 0.25–12.5%, more than 9% of OMDT patients die due to secondary severe infection, multiorgan failure, and hepatic encephalopathy (5, 6). Patients who survive after high-dose glucocorticoid treatment are also often accompanied by multiple complications, such as hyperlipidemia, infection, and femoral head necrosis, and they can easily relapse. Due to its severity, OMDT has become one of the critical but unresolved issues in the field of dermatology and occupational health (7).

OMDT is currently considered to be a T-cell-mediated type IV hypersensitivity reaction. In addition to skin and mucosal lesions, OMDT patients are often accompanied by severe kidney injury. Clinical analysis indicates that renal injury in OMDT patients is characterized by diffused inflammation of both kidneys with slight accretion, cortical echo enhancement and densification, and renal insufficiency (8). Our epidemiological data also showed that urinary protein (PRO), serum creatinine (sCr) and blood urea nitrogen (BUN) levels were significantly elevated in OMDT patients compared with unaffected workers (9, 10). In addition, the results of our TCE-sensitized mouse model also showed that the sCr and BUN levels of TCE-sensitized-positive mice were significantly higher than those in control and TCE-sensitized-negative mice (10, 11). Pathological examination showed that the renal tubules of TCE-sensitized-positive mice presented with obvious vacuolar degeneration and infiltration of inflammatory cells (12). However, the exact mechanism of how TCE sensitization causes kidney injury remains unclear.

Our recent study shows that C5b-9-dependent cytosolic Ca^2+^ overload-induced renal tubular ferroptosis plays an important role in TCE sensitization-induced kidney injury, while CD59, a complement regulator protein, and ferrostatin-1 (Fer-1, a ferroptosis inhibitor) treatment can block renal tubular ferroptosis caused by TCE sensitization (13). This raises a new questions as to the causes of cytosolic Ca^2+^ overload and the specific mechanism that mediates ferroptosis in renal tubular epithelial cells of TCE-sensitized positive. C5b-9, the terminal complement activation product, can be assembled by complement C5b6 and plasma proteins C7-C9 on the surface of the cell membrane to damage target cells. Our recent studies have consistently demonstrated that C5b-9 is specifically deposited in the renal tubules of TCE-sensitized mice and that exogenous supplementation with CD59 attenuated renal tubular injury in TCE-sensitized positive mice (14–16). A previous study reports that the activation of inositol 1,4,5-triphosphate receptor (IP3R) caused by the complement membrane attack complex triggers endoplasmic reticulum Ca^2+^ release into the cytoplasm to increase the cytosolic Ca^2+^ level and result in cellular inflammatory responses in lung epithelial cells (17). Cytosolic Ca^2+^ rises caused by endoplasmic reticulum Ca^2+^ release or Ca^2+^ influx from the plasma membrane can all favor mitochondrial Ca^2+^ intake by activating the mitochondrial calcium uniporter (MCU) (18, 19). Luongo et al. (20) show that mitochondrial Ca^2+^ overload causes mitochondrial permeability transition pore (mPTP) opening, leading to increased superoxide production and necrotic cell death.

Many investigators have confirmed that mitochondrial stress and dysfunction increase the sensitivity of cells to ferroptosis through mitochondrial Fe^2+^ disturbance and mitochondrial reactive oxygen species (ROS) (21, 22). In addition, mitochondrial Ca^2+^ disorder has been shown to play an important role in cold stress-induced lipid peroxidation and ferroptosis (23). However, the role of IP3R-dependent mitochondrial disorder in ferroptosis of TCE-sensitized-positive mouse tubular cells and C5b-9-attacked HK-2 pathological model cells remains unclear. The purpose of our study was to explore the role of IP3R-dependent mitochondrial dysfunction in C5b-9 mediated renal tubular ferroptosis of trichloroethylene sensitization positive mice using TCE transdermal sensitization mouse model and C5b-9-attacked cell model.

## 2 Materials and methods

### 2.1 Animal experiments

**Figure 1:**
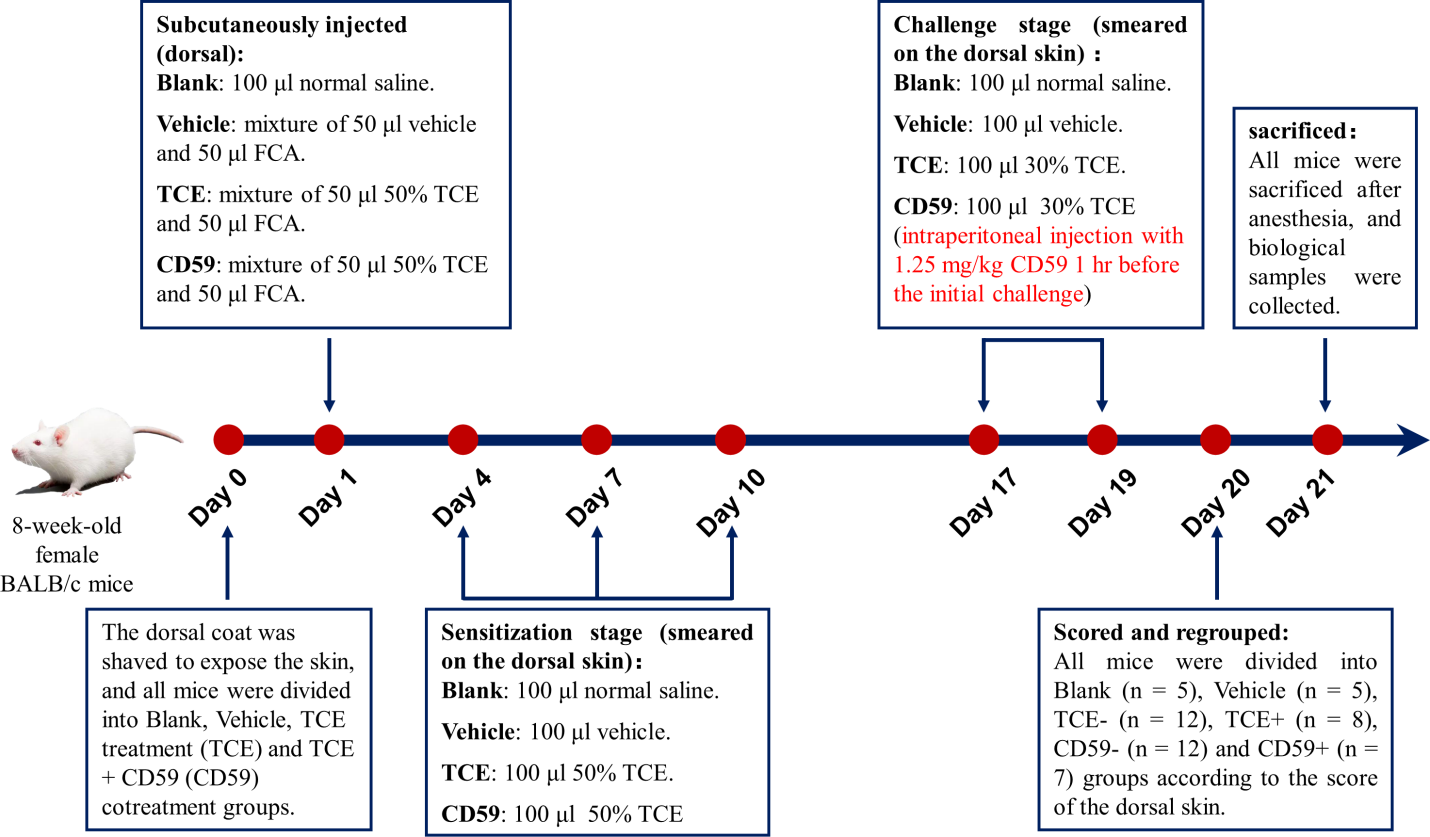
Model of TCE-sensitized mice. Eight-week-old female BALB/c mice were shaved of the dorsal hair to expose the dorsal skin 24 hr before the first treatment. On the first day, mice were subcutaneously injected with a mixture of 50 μl 50% TCE (TCE:olive oil:acetone = 5:2:3, v:v:v) and 50 μl Freund’s complete adjuvant (FCA, Sigma, Cat. F5881). On the 4th, 7th, and 10th days, 100 μl 50% TCE was applied to the dorsal skin of the mice. On days 17 and 19, 100 μl 30% TCE (TCE:olive oil:acetone = 3:2:5, v:v:v) was applied to the dorsal skin of the mice with or without pretreatment with CD59, a C5b-9 inhibitory protein. On day 20, all mice were divided into Blank group, Vehicle group, TCE sensitization negative group (TCE-), TCE sensitization positive group (TCE+), TCE+CD59 sensitization negative group (CD59-) and TCE+CD59 sensitization positive group (CD59+) according to the score of the dorsal skin.

Eight-week-old female BALB/c mice were purchased from the Experimental Animal Center of Anhui (Anhui, China). The TCE transcutaneous sensitization mouse model mimicking the state of OMDT patients was established as we previously described (11). All mice were divided into the blank control group (Blank), vehicle control group (Vehicle), TCE treatment group (TCE), and TCE+CD59 cotreatment group (CD59) 24 hr before treatment, and the dorsal hair was shaved to expose the dorsal skin. Details of the TCE-sensitized mouse model are shown in **Figure 1**. All animal experiments were conducted in accordance with the “Administrative Regulations on Laboratory Animals” issued by the Ministry of Science and Technology of the People’s Republic of China and approved by Anhui Medical University (Animal Ethics Committee No.: 20210897).

### 2.2 Extraction of primary tubular epithelial cells

Mouse renal tubular epithelial cells were isolated and cultured as we previously described (13). Briefly, fresh renal cortex was diced into pieces of approximately 1 mm^3^ after the mice were sacrificed. Tissue fragments were digested in 5 ml of DMEN/F12 serum-free medium containing 1 mg/ml collagenase and 1% penicillin-streptomycin solution at 37°C for 30 min. After digesting the tissue fragments, the cell suspension was poured through a nylon sieve with 70 μm pore. The suspension was centrifuged at 1000 RPM for 8 min, and the supernatant was discarded. Then, the cell clump was washed with 4 mL PBS and cultured in DMEM/F-12 medium supplemented with 10% mouse’s own serum at 37°C and 5% CO_2_.

### 2.3 Cell culture

Human renal proximal tubular epithelial cells (HK-2 cells) purchased from Fuheng Biotechnology (Shanghai, China) were cultured in DMEM/F12 medium (Gibco, Cat. 11320033) supplemented with 10% fetal bovine serum (FBS, Biological Industries, Cat. 04-001-1A) and 1% penicillin-streptomycin solution (Beyotime, Cat. C0222) at 37°C in a humidified atmosphere containing 5% CO2. Complement C5b6 (Complement Technology, Cat. A122) and normal human serum (NHS) were used to assemble C5b-9 on the cytomembrane according to a previous report (24). To explore the underlying mechanism whereby C5b-9 induces ferroptosis, HK-2 cells were treated with *IP3R* siRNA, 2 μM BAPTA-AM (Selleck, Cat. S7534) or 10 nM cyclosporin A (CsA, Selleck, Cat. S2286).

### 2.4 Cell viability assay

HK-2 cells were seeded into 96-well cell plates, and the cell viability was detected using a CCK8 kit (Beyotime, Cat. C0037) as the suppliers’ manual.

### 2.5 RNA interference of IP3R

Human IP3R-specific small interfering RNA (siR, GenePharma) and lipofectamine 3000 (ThermoFisher Scientific, Cat. L3000015) were mixed in serum-free DMEM/F-12 medium for 15 min. Then, the mixture was added to the culture medium to transfect HK-2 cells for 6 h. After this, the cells continued to be cultured in fresh medium for 48 h and treated with the test compounds. After washing, the cells were harvested and subjected to specific experiments. The sequences of IP3R siR were 5’-GCAGAUCUUCAAGUUGUUATT-3′ (forward) and 5’-UAACAACUUGAAGAUCUGCTT-3′ (reverse). The sequences of the negative control siR were 5’-UUCUCCGAACGUGUCACGUTT-3′ (forward) and 5’-ACGUGACACGUUCGGAGAATT-3′ (reverse).

### 2.6 Lipid peroxidation detection

Lipid peroxidation was detected by BODIPY™ 581/591 C11 (ThermoFisher Scientific, Cat. D3861). Briefly, HK-2 cells were incubated in serum-free DMEN/F-12 medium containing 10 μM BODIPY lipid probe for 30 min at 37°C and 5% CO_2_ after the test compounds were treated and subsequently counterstained with Hoechst 33342 (Beyotime, Cat. C1028). Then, the medium was removed, and the cells were washed 3 times with PBS protected from light. The oxidized lipid (green), non-oxidized lipid (red) and nuclei (blue) were imaged using a fluorescence microscope.

### 2.7 Analysis of mitochondrial membrane potential

Mitochondrial membrane potential was detected using an enhanced mitochondrial membrane potential assay kit with JC-1 (Beyotime, Cat. C2003S). Mitochondrial accumulation of JC-1 is membrane potential dependent and leads to the formation of red fluorescent J-aggregates. The mouse primary tubular epithelial cells or HK-2 cells were incubated with 10 μg/ml JC-1 probe for 20 min at 37°C and 5% CO_2_ after the test compounds were administered and subsequently counterstained with Hoechst 33342. The J-monomers (green), J-aggregates (red) and nuclei (blue) were imaged using a fluorescence microscope. Moreover, flow cytometry was also used to detect the mitochondrial membrane potential. Briefly, HK-2 cells were cultured in a 24-well plate treated with the test compounds and harvested by trypsinization. Then, cell clumps were resuspended in 500 μl serum-free DMEN/F-12 medium containing 10 μg/ml JC-1 probe and cultured at 37°C and 5% CO_2_ for 20 min. JC-1 monomers and aggregates were detected from the FITC and PE channels, respectively, using a flow cytometer.

### 2.8 Analysis of the mPTP

mPTP was detected using a mitochondrial permeability transition pore assay kit (Abcam, Cat. 239704). Mouse primary tubular epithelial cells or HK-2 cells were cultured in a 12-well plate treated with the test compounds and harvested by trypsinization. The cells were incubated in 1,000 μl pre-warmed mPTP wash buffer containing 2 μg/ml mPTP staining dye and 5 μl CoCl2 solution at 37°C and 5% CO2 for 15 min protected from light. Cells were centrifuged at 4°C and 1000 × g for 5 min after incubation. Then, the cells were resuspended in 1 mL of mPTP wash buffer to remove excess staining and quenching reagents. Fluorescence intensity was detected from the FITC channel using a flow cytometer.

### 2.9 Total protein extraction and immunoblotting

Renal cortex and HK-2 cells were homogenized in RIPA buffer (Beyotime, Cat, P0013B) and centrifuged at 15,000 × g and 4°C for 15 min to remove cell debris. A BCA protein assay kit (ThermoFisher Scientific, Cat. 23225) was used to detect the protein concentration. The total kidney protein was quantified as 3 μg/μl and the HK-2 cell protein was quantified as 2 μg/μl. The proteins were separated by SDS-PAGE electrophoresis and subsequently transferred to a polyvinylidene fluoride membrane. Membrane was incubated overnight at 4°C using the specific primary antibody after blocking 2 hr with 5% skimmed milk at room temperature. The specific primary antibodies include: anti-ACSL4 antibody (Santa Cruz, Cat. sc-365230, dilution 1:1,000), anti-COX2 antibody (Santa Cruz, Cat. sc-19999, dilution 1:1,000), anti-GPX4 antibody (Abcam, Cat. ab125066, dilution 1:2,000), anti-IP3R antibody (Santa Cruz, Cat. sc-398434, dilution 1:1,000), anti-MCU antibody (Santa Cruz, Cat. sc-515930, dilution 1:1,000), anti-OXPHOS antibody (Abcam, Cat. ab110413, dilution 1:5,000) and anti-GAPDH antibody (Abcam, Cat. ab8245, dilution 1:10,000). Then, the membrane was incubated with secondary antibodies for 2 hr at room temperature after washing with TBST buffer 4 times for 10 min each time. The WesternLumaxLight Sirius HRP substrate kit (Zeta Life, Cat. 310231) was used for color reaction. GAPDH was used as a loading control for total proteins. ImageJ software was used to quantify the protein band intensities (National Institutes of Health, Bethesda, MD, USA).

### 2.10 RNA extraction and real-time PCR

Total RNA was extracted from the renal cortex or HK-2 cells using TRIzol reagent (ThermoFisher Scientific, Cat. 15596026) and 1-bromine-3-chloropane (1-BCP, Sigma-Aldrich, Cat. B9673). The isolated RNA was reverse-transcribed to cDNA using the reverse transcription system (Promega Corporation, Cat. A3500) following the instruction manual. Real-time PCR was carried out using LightCycler^®^ 480 SYBR^®^ Green I Master (Roche, Cat. 04887352001). *Gapdh* served as a loading control, and the gene-specific primers are listed in **Table S1**.

### 2.11 Glutathione and malondialdehyde detection

The GSH levels in the renal cortex and HK-2 cells were detected using a GSH detection kit (Nanjing Jiancheng Bioengineering Institute, Cat. A006-2). The MDA levels in the renal cortex and HK-2 cells were detected using an MDA detection kit (Nanjing Jiancheng Bioengineering Institute, Cat. A003-1). GSH and MDA levels were normalized to per gram of protein.

### 2.12 Intracellular Fe^2+^ detection

Intracellular Fe^2+^ was detected using the Fe^2+^ detection probe FerroOrange (DOJINDO, Cat. F374). Briefly, HK-2 cells were incubated with Hank’s balanced salt solution (HBSS) containing 1 μM FerroOrange dye at 37°C and 5% CO_2_ for 30 min after the test compounds were applied. Cells were imaged immediately using a fluorescence microscope.

### 2.13 Cytosolic Ca^2+^ detection

Cytosolic Ca^2+^ was detected using a Fluo-8 calcium flux assay kit (Abcam, Cat. ab112129). HK-2 cells and mouse primary tubular epithelial cells were cultured with 100 μl Fluo-8 dye-loading solution at 37°C for 30 min and at room temperature for another 30 min in a black wall and clear bottom 96-well plate. The relative cytosolic Ca^2+^ level was detected at Ex/Em = 490/525 nm using a multimode plate reader (PerkinElmer).

### 2.14 Mitochondrial Ca^2+^ detection

Mitochondrial Ca^2+^ was detected with the mitochondrial Ca^2+^ fluorescent probe Rhod-2/AM probes (Yeasen, Cat. 40776ES72). Briefly, HK-2 cells were incubated with HBSS (without Ca^2+^ and Mg^2+^) containing 4 μM Rhod-2/AM dye at 37°C and 5% CO_2_ for 30 min after the test compounds were applied. Cells are washed 3 times with HBSS to adequately remove residual Rhod-2/AM working solution. Then, cells were incubated with in a 37°C incubator for another 30 min to ensure complete de-esterification of AM bodies within the cells. The MitoTracker Green probes were used to localize the mitochondrial. Cells were imaged immediately using a fluorescence microscope.

### 2.15 Statistical analysis

All statistical analyses were performed using SPSS 23.0 software (SPSS Inc., Chicago, USA). The mean values between different groups were compared using two-way ANOVA with LSD’s multiple comparison *post-hoc* test. All quantitative data are expressed as the means ± S.E.M. Differences were considered statistically significant at *P*<0.05.

## 3 Results

### 3.1 Sensitization rate of the TCE sensitization mouse model

TCE- and CD59-group mice were divided into a sensitization-positive group (erythema or edema) and a sensitization-negative group (no reaction) according to the dorsal skin reaction (**Figure S1**). As shown in **Table 1**, the sensitization rate of the TCE group was 40.00% (8/20), and the sensitization rate of the TCE+CD59 treatment group was 36.84% (7/19). No obvious skin reaction was observed in Blank- and Vehicle-group mice.

**Table 1:** The sensitization rate of different group mice.

		Sample number (n)	Sensitization rate (%)
Blank control		5	–
Vehicle control		5	–
TCE	Negative	12	40.00
	Positive	8	
TCE+CD59	Negative	12	36.84
	Positive	7	

### 3.2 Deposition of C5b-9 activated IP3R

**Figure 2:**
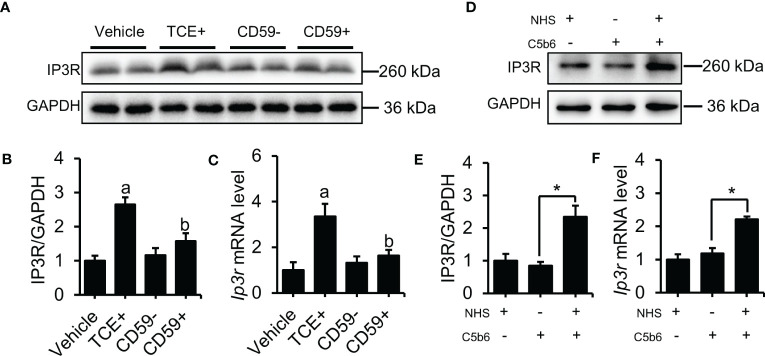
Deposition of C5b-9 activated IP3R. **(A)** IP3R protein in the mouse renal cortex was detected using immunoblotting. **(B)** Quantitative analysis of IP3R protein levels. **(C)** *Ip3r* mRNA levels in the mouse renal cortex were measured using RT-PCR. **(D)** IP3R protein expression in HK-2 cells was detected using immunoblotting. **(E)** Quantitative analysis of IP3R protein levels. **(F)** *Ip3r* mRNA levels in HK-2 cells were measured using RT-PCR. Quantitative data are shown as the mean ± SEM. (n = 4-6 per group). ^a^ *P* < 0.05 compared with the Vehicle group, ^b^ *P* < 0.05 compared with the TCE+ group, *P < 0.05.

Our previous results show that C5b-9 increases the cytosolic Ca^2+^ level in TCE-sensitization-positive mouse primary tubular epithelial cells and C5b-9-attacked HK-2 cells, but the underlying mechanism remains unclear. Here, we detected the upregulation of IP3R, an endoplasmic reticulum calcium release channel protein, using immunoblotting and RT-PCR methods. As expected, the IP3R protein and mRNA level was significantly higher in the TCE+ group than in the Blank, Vehicle and TCE- groups (**Figures S2A, B**), while pretreatment with CD59 reversed the abnormal increase of the IP3R protein and mRNA in the renal tubules of TCE sensitization-positive mice (**Figures 2A–C**). To explore the role of C5b-9 in the upregulation of IP3R, we assembled C5b-9 on the cytomembrane of HK-2 cells as described in our previous study (13). Immunofluorescence results showed that C5b-9 was assembled after C5b6 and NHS co-treatment (**Figure S3**). Interestingly, IP3R was clearly upregulated by C5b-9, as IP3R protein and mRNA levels in HK-2 cells were markedly increased after C5b-9 was assembled (**Figures 2D–F**). The consistent changes in mRNA levels support the IP3R gene expression underlying the increased IP3R protein levels. These results suggest that the cytosolic Ca^2+^ overload caused by TCE sensitization may be due to an upregulation of IP3R.

### 3.3 IP3R upregulation induced cytosolic Ca^2+^ overload and mitochondrial dysfunction in TCE-sensitized mouse kidneys and HK-2 cells

**Figure 3:**
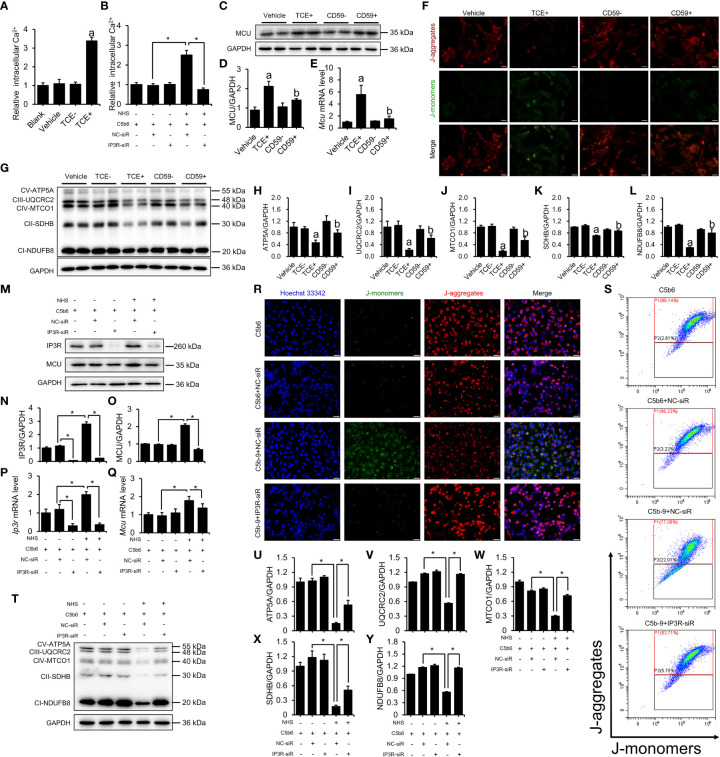
IP3R activation induced cytosolic Ca^2+^ overload and mitochondrial dysfunction. The HK-2 cells were cultured with C5b6 and NHS to assemble C5b-9 on the cytomembrane after *IP3R* siR was transfected. **(A)** Relative cytosolic Ca^2+^ concentration of TCE-sensitized mouse primary tubular epithelial cells was detected by monitoring the fluorescence intensity at Ex/Em = 490/525 nm using a multimode plate reader (PerkinElmer). **(B)** The relative cytosolic Ca^2+^ concentration of HK-2 cells was detected. **(C)** MCU protein in the mouse renal cortex was detected using immunoblotting. **(D)** Quantitative analysis of MCU protein levels. **(E)** *Mcu* mRNA levels in the mouse renal cortex were measured using RT-PCR. **(F)** The mitochondrial membrane potential of TCE-sensitized mouse primary tubular epithelial cells was detected using JC-1 dye. The scale bar represents 50 μm. **(G)** The essential components of OXPHOS in the mouse renal cortex were detected using immunoblotting. **(H–L)** Quantitative analysis of the essential components of OXPHOS. **(M)** IP3R and MCU proteins of HK-2 cells were detected using immunoblotting. **(N, O)** Quantitative analysis of IP3R and MCU protein levels. **(P, Q)** *Ip3r* and *Mcu* mRNA levels in HK-2 cells were measured using RT-PCR. **(R)** The mitochondrial membrane potential of HK-2 cells was detected using JC-1 dye. The scale bar represents 50 μm. **(S)** Flow cytometry results showing the mitochondrial membrane potential of HK-2 cells. **(T)** The essential components of OXPHOS in HK-2 cells were detected using immunoblotting. **(U–Y)** Quantitative analysis of the essential components of OXPHOS. Quantitative data are shown as the mean ± SEM. (n = 4-6 per group). ^a^ *P* < 0.05 compared with the Vehicle group, ^b^ *P* < 0.05 compared with the TCE+ group, *P < 0.05.

To explore the potential mechanism whereby C5b-9 induces renal tubule ferroptosis, we examined Ca^2+^ levels and mitochondrial damage in tubular epithelial cells from TCE-sensitized mouse kidneys and C5b-9-attacked HK-2 cells. Mouse primary tubular epithelial cells were extracted and cultured with 10% of their own sera, and immunofluorescence was used to identify tubular epithelial cells. CK-18 and Nephrin were used to mark tubular epithelial cells and podocytes, respectively (**Figure S4A**). **Figure 3A** shows that the Ca^2+^ concentration was significantly higher in primary tubular epithelial cells of TCE+ group mice than in Blank, Vehicle and TCE- group mice. C5b-9 inhibition markedly reduced the increased cytosolic Ca^2+^ level by TCE sensitization (**Figure S5A**). To explore the role of C5b-9 in cytosolic Ca^2+^ overload in HK-2 cells, we used NHS and 0.2 μg/ml C5b6 to assemble C5b-9 on the surface of HK-2 cells (**Figure S3**). As expect, the relative cytosolic Ca^2+^ levels of C5b-9-attacked HK-2 cells were higher than those of normal cells (**Figure 3B**). The mitochondrial calcium uniporter (MCU) protein and mRNA was increased in the kidneys of TCE+ group mice, and CD59 alleviated the abnormal upregulation (**Figures S2A, C**; **3C–E**). Consistently, the mitochondrial membrane potential of primary tubular epithelial cells of TCE+ group mice was decreased, as seen by the increased JC-1 monomers fluorescence intensity (**Figure S4B**). Meanwhile, immunoblotting analysis showed that the essential components of oxidative phosphorylation system (OXPHOS) was significant lower in TCE sensitization-positive mice as compared to Vehicle and TCE sensitization negative mice (**Figures 3G–L**). Interestingly, mitochondrial membrane potential depolarization and the decrease in the essential components of OXPHOS induced by TCE sensitization were markedly ameliorated by CD59 pretreatment (**Figures 3F–L**). These data provide experimental evidence that TCE sensitization induced C5b-9 deposition causes IP3R upregulation, cytosolic Ca^2+^ overload, mitochondrial calcium influx and dissipation of mitochondrial membrane potential and consequent loss of the mitochondrial function - oxidative phosphorylation.

The key role of IP3R was also verified in HK-2 cells. To further investigate the role of IP3R in C5b-9-induced Ca^2+^ overload and mitochondrial dysfunction, *IP3R* siR was used in HK-2 cells before C5b-9 assembled. RT-PCR was used to detect the effectiveness of IP3R RNA interference (**Figure S5B**). As expected, *IP3R* siR pretreatment reduced C5b-9-induced increase in cytosolic Ca^2+^ levels in HK-2 cells (**Figure 3B**). Meanwhile, *IP3R* siR pretreatment normalized C5b-9-augmented IP3R and MCU protein and mRNA levels (**Figures 3M–Q**). In addition, to study the alteration of mitochondrial Ca^2+^ levels, we detected mitochondrial Ca^2+^ levels with the mitochondrial Ca^2+^ fluorescent probe Rhod-2 AM and the mitochondrial fluorescent probe MitoTracker Green. As shown in **Figure S5C**, C5b-9 significantly cause an increase in mitochondrial Ca^2+^ levels, while pretreatment with *IP3R* siR alleviated the abnormal changes in mitochondrial Ca^2+^ caused by C5b-9. Mitochondrial membrane potential was analyzed by fluorescence microscopy and flow cytometry after staining with JC-1 dye. As shown in **Figures 3R, S** and **Figure S6A**, *IP3R* siR pretreatment markedly counteracted C5b-9-induced mitochondrial membrane potential depolarization. In addition, *IP3R* siR effectively restored C5b-9-downregulated expression of the essential components of OXPHOS (**Figures 3T–Y**).

In summary, these results suggest that IP3R upregulation plays an important role in C5b-9-induced Ca^2+^ overload and mitochondrial damage in the renal tubules of TCE sensitization-positive mice and HK-2 cells.

### 3.4 IP3R inhibition protected HK-2 cells from ferroptosis induced by C5b-9 deposition

**Figure 4:**
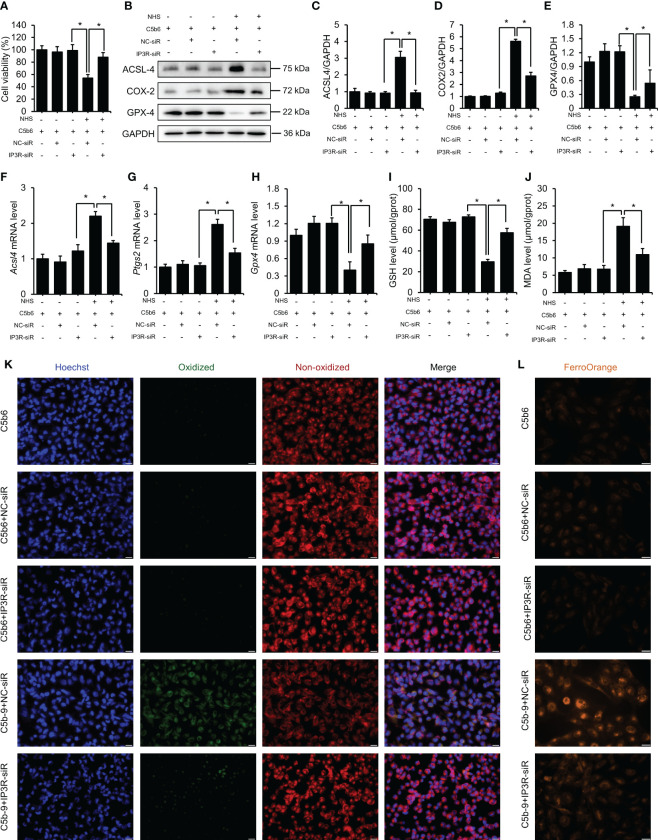
IP3R siR protected HK-2 cells from ferroptosis induced by C5b-9. The HK-2 cells were cultured with C5b6 and NHS to assemble C5b-9 on the cytomembrane after *IP3R* siR was transfected. **(A)** Cell viability of HK-2 cells after 24 hr of C5b-9 assembly. **(B)** ACSL4, COX2 and GPX4 proteins in HK-2 cells were detected using immunoblotting after treatment with *IP3R* siR. **(C–E)** Quantitative analysis of ACSL4, COX2 and GPX4 proteins. **(F–H)** *Acsl4*, *Ptgs2* and *Gpx4* mRNA levels in HK-2 cells were measured using RT-PCR after treatment with *IP3R* siR. **(I)** GSH levels in HK-2 cells after treatment with siR. **(J)** MDA level of HK-2 cells after treatment with *IP3R* siR. **(K)** Lipid ROS were detected by BODIPY™ 581/591 C11 after treatment with *IP3R* siR. The green fluorescence represents oxidized lipids, and the red fluorescence represents non-oxidized lipids. The scale bar represents 50 μm. **(L)** Fe^2+^ was detected by FerroOrange dye. The scale bar represents 50 μm. Quantitative data are shown as the mean ± SEM. (n = 4-6 per group). *P < 0.05.

To explore whether the ferroptosis of HK-2 cells induced by C5b-9 was caused by IP3R upregulation, we detected the expression of ferroptosis-related indicators after *IP3R* siR pretreatment. As **Figure 4A** shows, *IP3R* siR abolished the cell death induced by C5b-9 in HK-2 cells. CoA synthetase long-chain family member 4 (ACSL4), an enzyme that converts fatty acids to fatty acyl-CoA esters, regulates lipid biosynthesis and contributes to ferroptosis. Additionally, cyclooxygenase-2 (COX2) and glutathione peroxidases 4 (GPX4) have been shown to participate in ferroptosis by regulating the cellular redox status. Our previous study showed that C5b-9 caused markedly abnormal changes in ACSL4, COX2 and GPX4 protein and mRNA levels in HK-2 cells. Interestingly, *IP3R* siR effectively reduced the C5b-9-increased ACSL4 and COX2 protein levels (**Figures 4B–D**). Furthermore, the C5b-9-suppressed GPX4 protein level was also clearly reversed by *IP3R* siR treatment (**Figures 4B, E**). Consistently, the C5b-9-augmented mRNA levels of *Acsl4* and *Ptgs2*, the encoding gene of COX2, were also antagonized by *IP3R* siR, while the decreased *Gpx4* mRNA level was blocked by *IP3R* siR (**Figures 4F–H**). GSH and MDA play an important role in maintaining the balance of oxidation and reduction, which was disrupted in C5b-9-attacked HK-2 cells. Further investigation showed that *IP3R* siR pretreatment attenuated C5b-9-induced aberrant changes in GSH and MDA levels (**Figures 4I, J**). The accumulation of lipid peroxide and intracellular iron metabolism disorders are other characteristics of ferroptosis and were detected by the BODIPY™ 581/591 C11 probe and FerroOrange dye, respectively. As shown in **Figure 4K**, the increase in lipid peroxide induced by C5b-9 was antagonized by *IP3R* siR. Finally, *IP3R* siR alleviated the upregulated Fe^2+^ level induced by C5b-9 (**Figure 4L**). In conclusion, these data show that IP3R activation causes lipid peroxidation and iron deposition and hence ferroptosis and suggest that the ferroptosis induced by C5b-9 likely occurs through activation of IP3R.

### 3.5 Cytosolic Ca^2+^ overload caused mPTP activation and mitochondrial dysfunction in HK-2 cells

**Figure 5:**
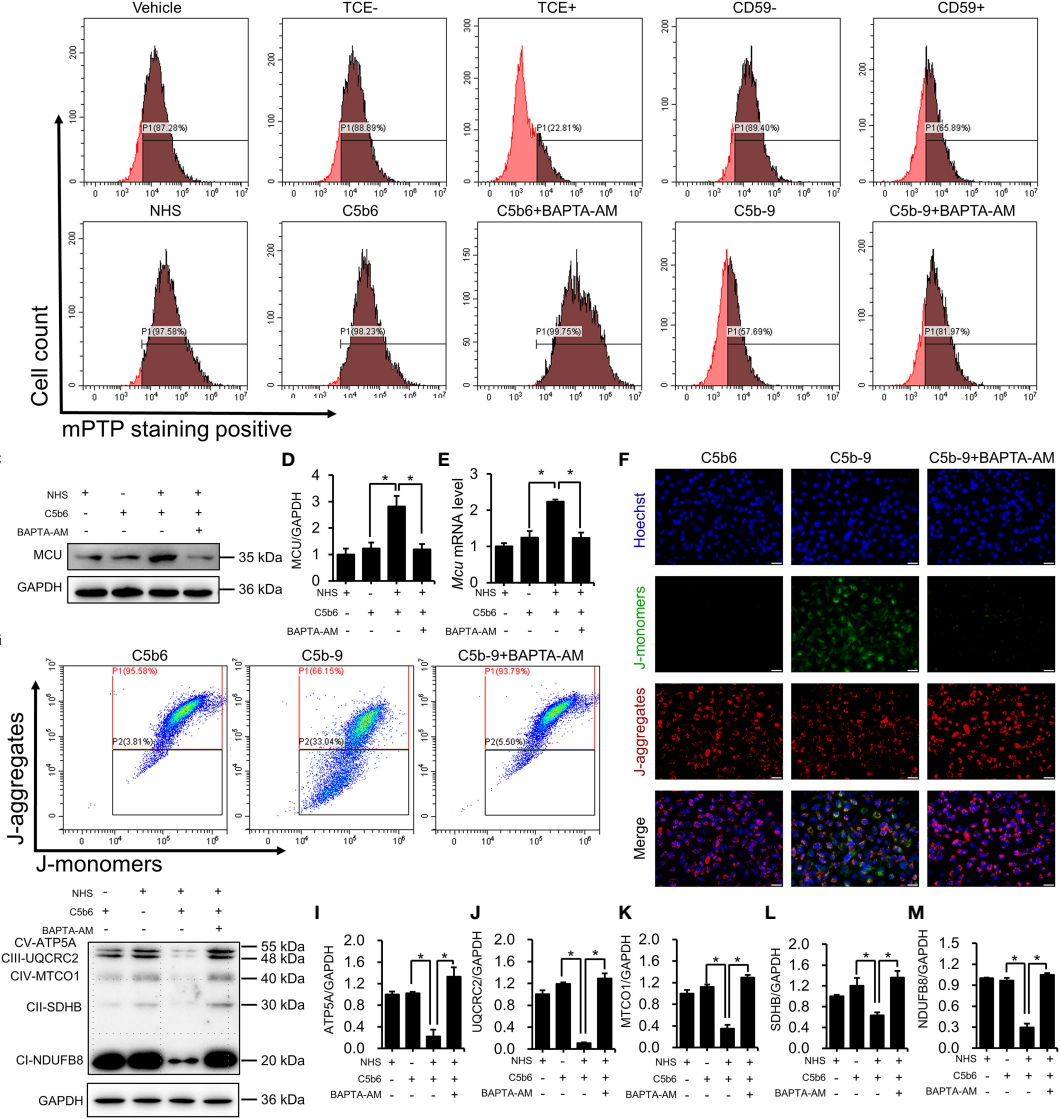
Cytosolic Ca^2+^ overload causes mPTP opening and mitochondrial dysfunction. **(A)** mPTP of TCE-sensitized mouse primary tubular epithelial cells was detected by mPTP staining dye. **(B)** mPTP of HK-2 cells was detected by mPTP staining dye. **(C)** MCU protein in HK-2 cells was detected using immunoblotting. **(D)** Quantitative analysis of MCU protein levels. **(E)** *Mcu* mRNA levels in HK-2 cells were measured using RT-PCR. **(F)** The mitochondrial membrane potential of HK-2 cells was detected using JC-1 dye. The scale bar represents 50 μm. **(G)** Flow cytometry results showing the mitochondrial membrane potential of HK-2 cells. **(H)** The essential components of OXPHOS in HK-2 cells were detected using immunoblotting. **(I–M)** Quantitative analysis of the essential components of OXPHOS. Quantitative data are shown as the mean ± SEM. (n = 4-6 per group). *P < 0.05.

To investigate the role of cytosolic Ca^2+^ overload in mitochondrial damage, we treated C5b-9-attacked HK-2 cells with BAPTA-AM, an intracellular Ca^2+^ chelator. The mPTP is a non-specific channel formed by components of the inner and outer mitochondrial membranes. Continuous mPTP opening causes subsequent loss of mitochondrial membrane potential. mPTP opening in primary tubular epithelial cells and HK-2 cells was detected using mPTP staining dye. As shown in **Figure 5A** and **S6B**, the mPTP opening was observed in the TCE+ group, while CD59 pretreatment alleviated this abnormal activity. Consistently, the mPTP was also opened in C5b-9-attacked HK-2 cells, and BAPTA-AM effectively rescued the cells from mPTP opening (**Figures 5B** and **S6C**). Immunoblotting analysis showed that BAPTA-AM reduced IP3R activation-augmented MCU protein and mRNA levels (**Figures 5C–E**). In addition, fluorescence imaging and flow cytometry also demonstrated that BAPTA-AM pretreatment antagonized the loss of mitochondrial membrane potential of HK-2 cells caused by IP3R activation(**Figures 5F, G**; **S6D**). Furthermore, the IP3R-mediated decrease in the essential components of OXPHOS was alleviated by BAPTA-AM (**Figures 5H–M**). Collectively, these results suggest that IP3R causes mPTP opening and mitochondrial dysfunction in TCE-sensitized mice and HK-2 cells and that cytosolic Ca^2+^ overload may play a key role.

### 3.6 mPTP inhibition alleviated IP3R-induced mitochondrial dysfunction

**Figure 6:**
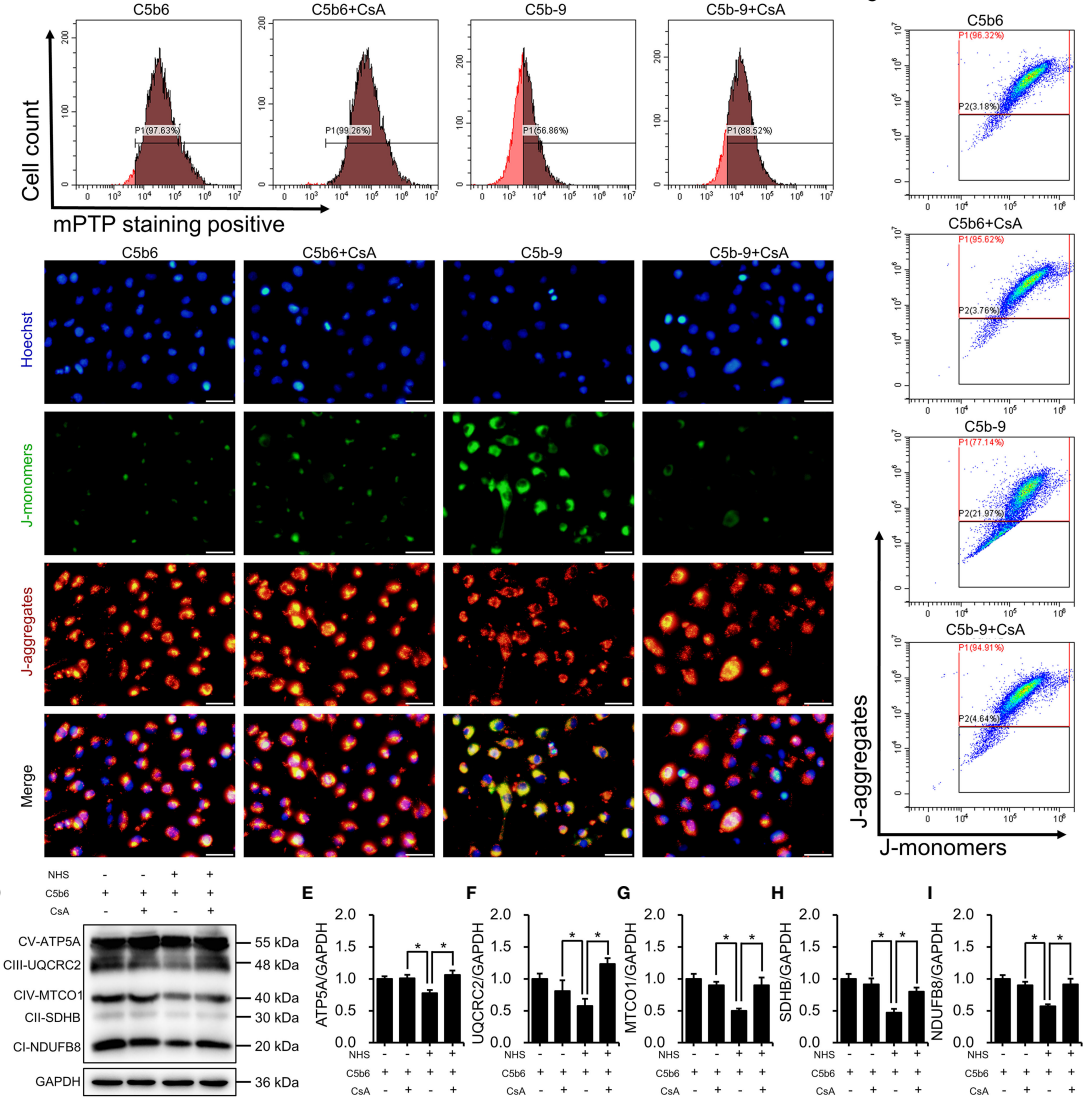
mPTP inhibition alleviated IP3R-induced mitochondrial dysfunction in HK-2 cells. **(A)** The mPTP of HK-2 cells was detected by mPTP staining dye. **(B)** The mitochondrial membrane potential of HK-2 cells was detected using JC-1 dye. The scale bar represents 50 μm. **(C)** Flow cytometry results showing the mitochondrial membrane potential of HK-2 cells. **(D)** The essential components of OXPHOS in HK-2 cells were detected using immunoblotting. **(E–I)** Quantitative analysis of the essential components of OXPHOS. Quantitative data are shown as the mean ± SEM. (n = 4-6 per group). *P < 0.05.

To investigate the role of mPTP activation in IP3R-induced mitochondrial dysfunction, CsA, a mPTP inhibitor, was used in HK-2 cells. The effectiveness of CsA was detected. As expected, CsA pretreatment clearly inhibited IP3R activation-induced mPTP opening (**Figure 6A**; **S6E**). Mitochondrial membrane potential was analyzed by fluorescence microscopy and flow cytometry after staining with JC-1 dye. As shown in **Figures 6B, C**; **S6F**, mPTP inhibition markedly counteracted IP3R activation-induced mitochondrial membrane potential depolarization. Next, the effect of CsA inhibition on the essential components of OXPHOS was analyzed. Interestingly, CsA inhibition effectively restored IP3R activation-downregulated expression of the essential components of OXPHOS (**Figures 6D–I**). In summary, these results suggest that mPTP opening plays an important role in IP3R activation-induced mitochondrial damage in HK-2 cells.

### 3.7 mPTP inhibition protected HK-2 cells from ferroptosis

**Figure 7:**
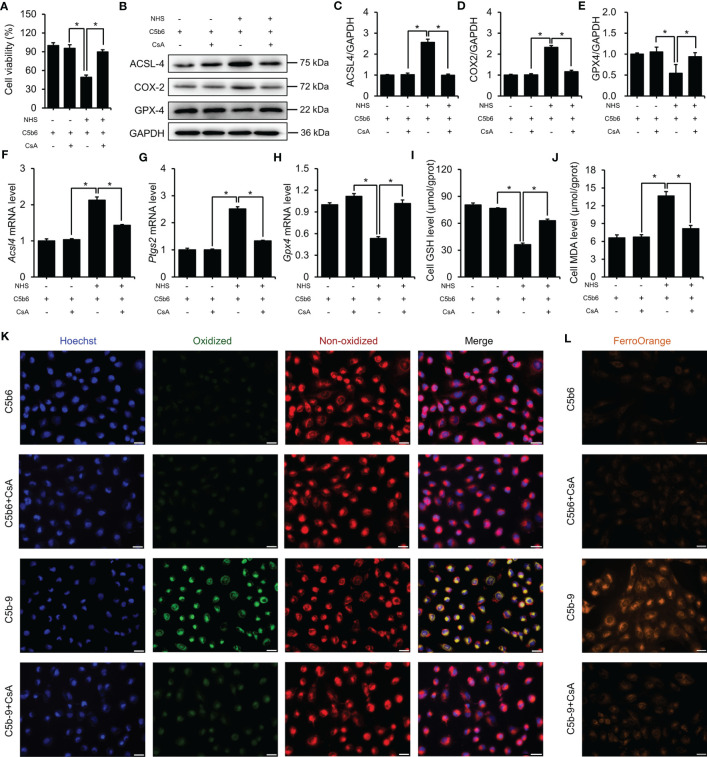
mPTP inhibition protected HK-2 cells from ferroptosis induced by C5b-9. **(A)** Cell viability of HK-2 cells. **(B)** ACSL4, COX2 and GPX4 proteins in HK-2 cells were detected using immunoblotting. **(C–E)** Quantitative analysis of ACSL4, COX2 and GPX4 proteins. **(F–H)** *Acsl4*, *Ptgs2* and *Gpx4* mRNA levels in HK-2 cells were measured using RT-PCR. **(I)** GSH levels in HK-2 cells. **(J)** MDA level of HK-2 cells. **(K)** Lipid ROS were detected by BODIPY™ 581/591 C11. The green fluorescence represents oxidized lipids, and the red fluorescence represents non-oxidized lipids. The scale bar represents 50 μm. **(L)** Fe^2+^ was detected by FerroOrange dye. The scale bar represents 50 μm. Quantitative data are shown as the mean ± SEM. (n = 4-6 per group). *P < 0.05.

The counter effect of mPTP inhibition by CsA on ferroptosis in a C5b-9-attacked HK-2 cell model was examined. Treatment with CsA significantly mitigated the C5b-9-induced decrease in cell viability (**Figure 7A**). Immunoblotting analysis showed that the defective expression of ferroptosis-associated proteins, such as ACSL4, COX2 and GPX4, was clearly rescued by pretreatment with CsA (**Figures 7B–E**). Consistently, the abnormal changes in *Acsl4*, *Ptgs2* and *Gpx4* mRNA levels were also counteracted after CsA treatment (**Figures 7F–H**). In addition, treatment with CsA markedly mitigated the IP3R activation-induced decrease in GSH levels and increase in MDA levels (**Figures 7I, J**). Furthermore, the BODIPY™ 581/591 C11 probe was used to detect the lipid peroxidation level. Fluorescence imaging showed that the oxidized lipid level was significantly decreased after CsA treatment (**Figure 7K**). To explore the intracellular free iron level, Fe^2+^ was measured using FerroOrange dye. As shown in **Figure 7L**, mPTP inhibition markedly alleviated intracellular free Fe^2+^ levels. Taken together, these results suggest that the activation of mPTP plays a key role in IP3R activation-induced ferroptosis of HK-2 cells.

## 4 Discussion

Our recent study confirmed the important role of renal tubular ferroptosis in TCE-induced immune kidney injury (13). We found that both ferroptosis inhibitor and exogenous C5b-9 regulatory protein attenuated not only TCE sensitization-induced immune kidney injury, but also TCE sensitization-induced TECs ferroptosis (13). However, the underlying mechanism was not clearly explained. Here, we report that IP3R-dependent cytosolic Ca^2+^ overload and mitochondrial dysfunction are implicated in the immune kidney injury of TCE sensitization-positive mice. This study demonstrates that tubule-specific C5b-9 deposition activates the endoplasmic reticulum calcium release channel protein IP3R, causing endoplasmic reticulum Ca^2+^ release into the cytoplasm. Our further investigation reveals that the overloaded Ca^2+^ activates MCU and causes mPTP opening, resulting in mitochondrial dysfunction and renal tubular ferroptosis, and conversely these effects are antagonized by the complement regulator protein CD59. Moreover, the *in vitro* model system studies also reproduced these phenomena. Importantly, IP3R inhibition alleviated C5b-9-induced ferroptosis, Ca^2+^ overload and mitochondrial dysfunction in HK-2 cells, supporting a causal relationship and the sequence events leading to ferroptosis. Finally, the mPTP inhibitor, CsA, also blocked IP3R activation-induced mitochondrial dysfunction and ferroptosis in HK-2 cells, providing further proof. All these coherent data provide compelling evidence that C5b-9 induces renal tubular ferroptosis likely by stimulating IP3R-mediated cytosolic Ca^2+^ overload and consequent mitochondrial dysfunction. To our knowledge, this is the first identification of C5b-9 initiated and a series of IP3R-mediated signaling events leading to cell death in the form of ferroptosis in renal tubular epithelial cells. This mechanism not only explains TCE sensitization-induced renal tubule damage, but also serves as a down-stream mechanism for any pathological stimuli which activate the C5b-9 complex.

IP3R, the most ubiquitous intracellular Ca^2+^ release channel, releases Ca^2+^ from the intracellular Ca^2+^ store-the endoplasmic reticulum into the cytoplasm in response to IP3 binding (25, 26). IP3R is involved in a variety of basic biological functions, such as G-protein-coupled receptor signaling, cell division, differentiation, fertilization and cell death (27). The over-activation of IP3R by various cellular stimuli results in the release of Ca^2+^ from the endoplasmic reticulum into the cytoplasm and mitochondria (28), leading to the disruption of Ca^2+^ homeostasis, accompanied by endoplasmic reticulum stress and mitochondrial dysfunction (29–31). Triantafilou et al. (17) reported that under the action of the membrane attack complex, IP3R was activated and thus caused the release of Ca^2+^ from the endoplasmic reticulum to the cytoplasm, resulting in an increase in the cytosolic Ca^2+^ concentration in primary human lung epithelial cells. Consistently, our data also show that C5b-9 upregulates IP3R in both the renal tubular epithelial cells of TCE-sensitized mice and HK-2 model cells and thus causes Ca^2+^ release from the endoplasmic reticulum into the cytoplasm. Further investigation shows that the mitochondrial calcium uniporter MCU is upregulated as a result of increased cytosolic Ca^2+^, as evidenced by the decreased expression after BAPTA-AM treatment. Convincingly, IP3R inhibition by *siR* not only reduced the C5b-9-increased cytosolic Ca^2+^ level and MCU protein expression but also antagonized C5b-9-induced ferroptosis. These findings suggest a multi-step signaling pathway that in renal tubular epithelial cells, C5b-9 increases the cytosolic Ca^2+^ level by activating IP3R, and cytosolic Ca^2+^ may enter mitochondria through MCU, causing mitochondrial dysfunction and ultimate ferroptosis. This is the first demonstration of an IP3-initiated pathway eventually leading to lipid peroxidation and ferroptosis in renal tubular epithelial cells. The IP3-mediated pathway leading to ferroptosis represents a novel mechanism for TCE-sensitization induced renal tubular damage.

The mPTP is a non-specific channel formed by components of the inner and outer mitochondrial membranes. In healthy cells, the mPTP is constantly switching between opened and closed states. However, under certain pathological conditions, the mPTP can dramatically alter mitochondrial permeability (32). Under physiological conditions, adequate Ca^2+^ transfer to mitochondria is essential for mitochondrial functionality and does not induce the opening of the mPTP. However, the excessive mitochondrial Ca^2+^ uptake stimulates mPTP opening, causing the loss of mitochondrial membrane potential and ultimate cell death (19). Our results showed that TCE sensitization and the C5b-9 assembly induced cytosolic Ca^2+^ rise in tubular epithelial cells caused mPTP opening and loss of mitochondrial membrane potential. Basit et al. (33) previously reported that mPTP opening and loss of mitochondrial membrane potential stimulated an increase in the associated ROS, leading to activation of combined necroptotic/ferroptotic cell death in melanoma cells. In addition, a recent study also showed that the mitochondrial Ca^2+^ homeostasis-mitochondrial membrane potential axis is involved in lipid peroxidation and ferroptosis in A549 cells (23). We show that mPTP is key to IP3 induced Ca^2+^ overload and ferroptosis in renal tubule cells. In our current study, mitochondrial dysfunction and ferroptosis were alleviated when mPTP was inhibited by CsA, suggesting that activation of mPTP plays a key role in IP3R activation-induced ferroptosis of HK-2 cells. The coherent and systemic data from our study identify a key role of ferroptosis in TCE sensitization-induced renal tubule lesions driven by IP3R-mediated cytosolic Ca^2+^ rise and mitochondrial dysfunction.

Although the present study provides sufficient evidence for the IP3R-dependent mitochondrial dysfunction-induced ferroptosis in renal tubular epithelial cells of TCE sensitization-positive mice, there are still some limitations. First of all, we only directly demonstrated that activation of IP3R by C5b-9 caused a rise in cytosolic Ca^2+^, but we did not examine changes in endoplasmic reticulum Ca^2+^ levels. Moreover, the effect of cytosolic Ca^2+^ rise on mitochondrial Ca^2+^ homeostasis was also not assessed. Endoplasmic reticulum and mitochondria-specific Ca^2+^ detection kits should be used to detect changes in endoplasmic reticulum and mitochondrial Ca^2+^ levels to demonstrate that mitochondrial Ca^2+^ homeostasis has been destroyed or Ca^2+^ imaging technology should be used to track the real-time changes of Ca^2+^ concentration. Secondly, we have yet to provide direct evidence that mitochondrial membrane potential depolarization can induce lipid peroxidation and ferroptosis in renal tubular cells, and this is the key point of our next study. Finally, tubular C5b-9 deposition and ferroptosis require further validation in OMDT patients.

In summary, we examined the role of IP3R upregulation in C5b-9-induced tubular epithelial cell ferroptosis in TCE sensitization-positive mice and the model system HK-2 cells. We demonstrate that the upregulation of IP3R induced by C5b-9 deposition causes cytosolic Ca^2+^ rise and mitochondrial dysfunction, and ultimately results in ferroptosis of tubular epithelial cells from TCE sensitization-positive mice. Furthermore, the *in vitro* model also reproduced these phenomena, and pretreatment with BAPTA-AM or CsA all alleviated IP3R upregulation-induced mitochondrial dysfunction and ferroptosis in HK-2 cells. These results explain the IP3R-mediated specific mechanism of ferroptosis in renal tubular cells of TCE-sensitized mice and identify ferroptosis as an effective therapeutic target for TCE-sensitized kidney injury.

## Data availability statement

The original contributions presented in the study are included in the article/**Supplementary Material**, further inquiries can be directed to the corresponding author/s.

## Ethics statement

The animal study was reviewed and approved by Anhui Medical University (Animal Ethics Committee No.: 20210897).

## Author contributions

ZL: Formal analysis, Data Curation, Writing - Original Draft. JM: Investigation, Data Curation. XLZ: Visualization. XSZ: Visualization. HX: Data curation. FW: Supervision. CW: Writing - Reviewing and Editing, Methodology, Funding acquisition. JZ: Writing - Reviewing and Editing, Data curation, Funding acquisition. QZ: Conceptualization, Validation, Funding acquisition. All authors contributed to the article and approved the submitted version.

## References

[1] Ilieva NM Wallen ZD De Miranda BR. Oral ingestion of the environmental toxicant trichloroethylene in rats induces alterations in the gut microbiome: relevance to idiopathic parkinson’s disease. Toxicol Appl Pharmacol (2022) 451:116176. doi: 10.1016/j.taap.2022.116176 35914559 PMC10581445

[2] Chiu WA Jinot J Scott CS Makris SL Cooper GS Dzubow RC. Human health effects of trichloroethylene: key findings and scientific issues. Environ Health Perspect (2013) 121(3):303–11. doi: 10.1289/ehp.1205879 PMC3621199 23249866

[3] Lash LH Chiu WA Guyton KZ Rusyn I. Trichloroethylene biotransformation and its role in mutagenicity, carcinogenicity and target organ toxicity. Mutat Res Rev Mutat Res (2014) 762:22–36. doi: 10.1016/j.mrrev.2014.04.003 25484616 PMC4254735

[4] Cichocki JA Guyton KZ Guha N Chiu WA Rusyn I Lash LH. Target organ metabolism, toxicity, and mechanisms of trichloroethylene and perchloroethylene: key similarities, differences, and data gaps. J Pharmacol Exp Ther (2016) 359:110–23. doi: 10.1124/jpet.116.232629 PMC5034707 27511820

[5] Watanabe H. Hypersensitivity syndrome due to trichloroethylene exposure: a severe generalized skin reaction resembling drug-induced hypersensitivity syndrome. J Dermatol (2011) 38:229–35. doi: 10.1111/j.1346-8138.2010.01155.x 21342224

[6] Nakajima T Wang H Yuan Y Ito Y Naito H Kawamoto Y. Increased serum anti-CYP2E1 IgG autoantibody levels may be involved in the pathogenesis of occupational trichloroethylene hypersensitivity syndrome: a case-control study. Arch Toxicol (2022) 96:2785–97. doi: 10.1007/s00204-022-03326-x PMC9352743 35763063

[7] Zhao N Song X Naito H Li H Huang Y Liu L. Trichloroethylene and trichloroethanol induce skin sensitization with focal hepatic necrosis in guinea pigs. J Occup. Health (2020) 62:e12142. doi: 10.1002/1348-9585.12142 32799435 PMC7428806

[8] Liu J. Clinical analysis of seven cases of trichloroethylene medicamentose-like dermatitis. Ind Health (2009) 47:685–8. doi: 10.2486/indhealth.47.685 19996547

[9] Wang F Dai Y Huang M Zhang C Huang L Wang H. Glomerular damage in trichloroethylene-sensitized mice: targeting cathepsin l-induced hyperactive mTOR signaling. Front Pharmacol (2021) 12:639878. doi: 10.3389/fphar.2021.639878 34393767 PMC8358928

[10] Xie H Wang H Wu Q Peng J Huang H Wang Y. Endothelin-1/Endothelin receptor type a-Angiopoietins/Tie-2 pathway in regulating the cross talk between glomerular endothelial cells and podocytes in trichloroethylene-induced renal immune injury. J Inflamm Res (2021) 14:761–76. doi: 10.2147/JIR.S301104 PMC7955787 33727850

[11] Wang H Zhang JX Li SL Wang F Zha WS Shen T. An animal model of trichloroethylene-induced skin sensitization in BALB/c mice. Int J Toxicol (2015) 34:442–53. doi: 10.1177/1091581815591222 26111540

[12] Zuo X Liu Z Ma J Ding Y Cai S Wu C. Wnt 5a mediated inflammatory injury of renal tubular epithelial cells dependent on calcium signaling pathway in trichloroethylene sensitized mice. Ecotoxicol. Environ Saf. (2022) 243:114019. doi: 10.1016/j.ecoenv.2022.114019 36030685 PMC12011277

[13] Liu Z Ma J Zuo X Zhang X Hong Y Cai S. C5b-9 mediates ferroptosis of tubular epithelial cells in trichloroethylene-sensitization mice. Ecotoxicol. Environ Saf. (2022) 244:114020. doi: 10.1016/j.ecoenv.2022.114020 36049330 PMC11957332

[14] Wang F Huang LP Dai YY Huang M Jiang W Ye LP. Terminal complement complex C5b-9 reduced megalin and cubilin-mediated tubule proteins uptake in a mouse model of trichloroethylene hypersensitivity syndrome. Toxicol Lett (2019) 317:110–9. doi: 10.1016/j.toxlet.2019.10.002 31618666

[15] Xie H Yang L Yang Y Jiang W Wang X Huang M. C5b-9 membrane attack complex activated NLRP3 inflammasome mediates renal tubular immune injury in trichloroethylene sensitized mice. Ecotoxicol. Environ Saf. (2021) 208:111439. doi: 10.1016/j.ecoenv.2020.111439 33039874

[16] Wang F Huang M Wang Y Hong Y Zang D Yang C. Membrane attack complex C5b-9 promotes renal tubular epithelial cell pyroptosis in trichloroethylene-sensitized mice. Front Pharmacol (2022) 13:877988. doi: 10.3389/fphar.2022.877988 35656289 PMC9152256

[17] Triantafilou K Hughes TR Triantafilou M Morgan BP. The complement membrane attack complex triggers intracellular Ca2+ fluxes leading to NLRP3 inflammasome activation. J Cell Sci (2013) 126:2903–13. doi: 10.1242/jcs.124388 23613465

[18] Filadi R Basso E Lefkimmiatis K Pozzan T. Beyond intracellular signaling: the ins and outs of second messengers microdomains. Adv Exp Med Biol (2017) 981:279–322. doi: 10.1007/978-3-319-55858-5_12 29594866

[19] Rossi A Pizzo P Filadi R. Calcium, mitochondria and cell metabolism: a functional triangle in bioenergetics. Biochim Biophys Acta Mol Cell Res (2019) 1866:1068–78. doi: 10.1016/j.bbamcr.2018.10.016 30982525

[20] Luongo TS Lambert JP Gross P Nwokedi M Lombardi AA Shanmughapriya S. The mitochondrial Na+/Ca2+ exchanger is essential for Ca2+ homeostasis and viability. Nature (2017) 545:93–7. doi: 10.1038/nature22082 PMC5731245 28445457

[21] Wei S Qiu T Yao X Wang N Jiang L Jia X. Arsenic induces pancreatic dysfunction and ferroptosis via mitochondrial ROS-autophagy-lysosomal pathway. J Haz Mater (2020) 384:121390. doi: 10.1016/j.jhazmat.2019.121390 31735470

[22] Gan B. Mitochondrial regulation of ferroptosis. J Cell Biol (2021) 220:e202105043. doi: 10.1083/jcb.202105043 34328510 PMC8329737

[23] Nakamura T Ogawa M Kojima K Takayanagi S Ishihara S Hattori K. The mitochondrial Ca2+ uptake regulator, MICU1, is involved in cold stress-induced ferroptosis. EMBO Rep (2021) 22:e51532. doi: 10.15252/embr.202051532 33822458 PMC8097382

[24] Adler S Baker PJ Johnson RJ Ochi RF Pritzl P Couser WG. Complement membrane attack complex stimulates production of reactive oxygen metabolites by cultured rat mesangial cells. J Clin Invest. (1986) 77:762–7. doi: 10.1172/JCI112372 PMC423461 3005365

[25] Garcia MI Boehning D. Casrdiac inositol 1,4,5-trisphosphate receptors. biochim. Biophys Acta Mol Cell Res (2017) 1864:907–14. doi: 10.1016/j.bbamcr.2016.11.017 PMC5511688 27884701

[26] Bartok A Weaver D Golenár T Nichtova Z Katona M Bánsághi S. IP3 receptor isoforms differently regulate ER-mitochondrial contacts and local calcium transfer. Nat Commun (2019) 10:3726. doi: 10.1038/s41467-019-11646-3 31427578 PMC6700175

[27] Egorova PA Bezprozvanny IB. Inositol 1,4,5-trisphosphate receptors and neurodegenerative disorders. FEBS J (2018) 285:3547–65. doi: 10.1111/febs.14366 29253316

[28] Cárdenas C Miller RA Smith I Bui T Molgó J Müller M. Essential regulation of cell bioenergetics by constitutive InsP3 receptor Ca2+ transfer to mitochondria. Cell (2010) 142:270–83. doi: 10.1016/j.cell.2010.06.007 PMC2911450 20655468

[29] Butler MR Ma H Yang F Belcher J Le YZ Mikoshiba K. Endoplasmic reticulum (ER) Ca2+-channel activity contributes to ER stress and cone death in cyclic nucleotide-gated channel deficiency. J Biol Chem (2017) 292:11189–205. doi: 10.1074/jbc.M117.782326 PMC5500788 28495882

[30] Xiao WC Zhang J Chen SL Shi YJ Xiao F An W. Alleviation of palmitic acid-induced endoplasmic reticulum stress by augmenter of liver regeneration through IP3R-controlled Ca(2+) release. J Cell Physiol (2018) 233:6148–57. doi: 10.1002/jcp.26463 29323715

[31] Namba T. BAP31 regulates mitochondrial function via interaction with Tom40 within ER-mitochondria contact sites. Sci Adv (2019) 5:eaaw1386. doi: 10.1126/sciadv.aaw1386 31206022 PMC6561740

[32] Angeli S Foulger A Chamoli M Peiris TH Gerencser A Shahmirzadi AA. The mitochondrial permeability transition pore activates the mitochondrial unfolded protein response and promotes aging. eLife (2021) 10:e63453. doi: 10.7554/eLife.63453 34467850 PMC8410078

[33] Basit F van Oppen LM Schöckel L Bossenbroek HM van Emst-de Vries SE Hermeling JC. Mitochondrial complex I inhibition triggers a mitophagy-dependent ROS increase leading to necroptosis and ferroptosis in melanoma cells. Cell Death Dis (2017) 8:e2716. doi: 10.1038/cddis.2017.133 28358377 PMC5386536

