# Supplementary material for: IP3R-dependent mitochondrial dysfunction mediates C5b-9-induced ferroptosis in trichloroethylene-caused immune kidney injury
Source: Front Immunol. 2023 Jun 13;14:1106693. doi: 10.3389/fimmu.2023.1106693 (PMC10294229; doi:10.3389/fimmu.2023.1106693)
Supplement: Supplementary file 1 [file DataSheet_1.docx]

**Table S1. Primers for real-time RT-PCR.**

| **Gene** | **Primer sequence (5’-3’)** | **Size (bp)** | **Species** |
| --- | --- | --- | --- |
| *Ip3r* | Forward: ATTTGTTCTCTGTATGCGGAGG | 140 | Mouse |
|  | Reverse: AGCTTAAAGAGGCAGTCTCTGA |  |  |
| *Mcu* | Forward: TGTGAGGCTACCCTCTCGAC | 140 | Mouse |
|  | Reverse: GAACGCCATCTGGTGAGTAGA |  |  |
| *Gapdh* | Forward: AACTTTGGCATTGTGGAAGG | 223 | Mouse |
|  | Reverse: ACACATTGGGGGTAGGAACA |  |  |
| *Acsl4* | Forward: CATCCCTGGAGCAGATACTCT | 96 | Human |
|  | Reverse: TCACTTAGGATTTCCCTGGTCC |  |  |
| *Ptgs2* | Forward: CTGGCGCTCAGCCATACAG | 94 | Human |
|  | Reverse: CGCACTTATACTGGTCAAATCCC |  |  |
| *Gpx4* | Forward: GAGGCAAGACCGAAGTAAACTAC | 100 | Human |
|  | Reverse: CCGAACTGGTTACACGGGAA |  |  |
| *Ip3r* | Forward: ATTGCTGGGGACCGTAATCC | 129 | Human |
|  | Reverse: TCCAATGTGACTCTCATGGCA |  |  |
| *Mcu* | Forward: AGGATCGGGGAATTGACAGAG | 216 | Human |
|  | Reverse: GTGTGGTGTATAGTTGCTGGAC |  |  |
| *Gapdh* | Forward: GGAGCGAGATCCCTCCAAAAT | 197 | Human |
|  | Reverse: GGCTGTTGTCATACTTCTCATGG |  |  |

Figure S1


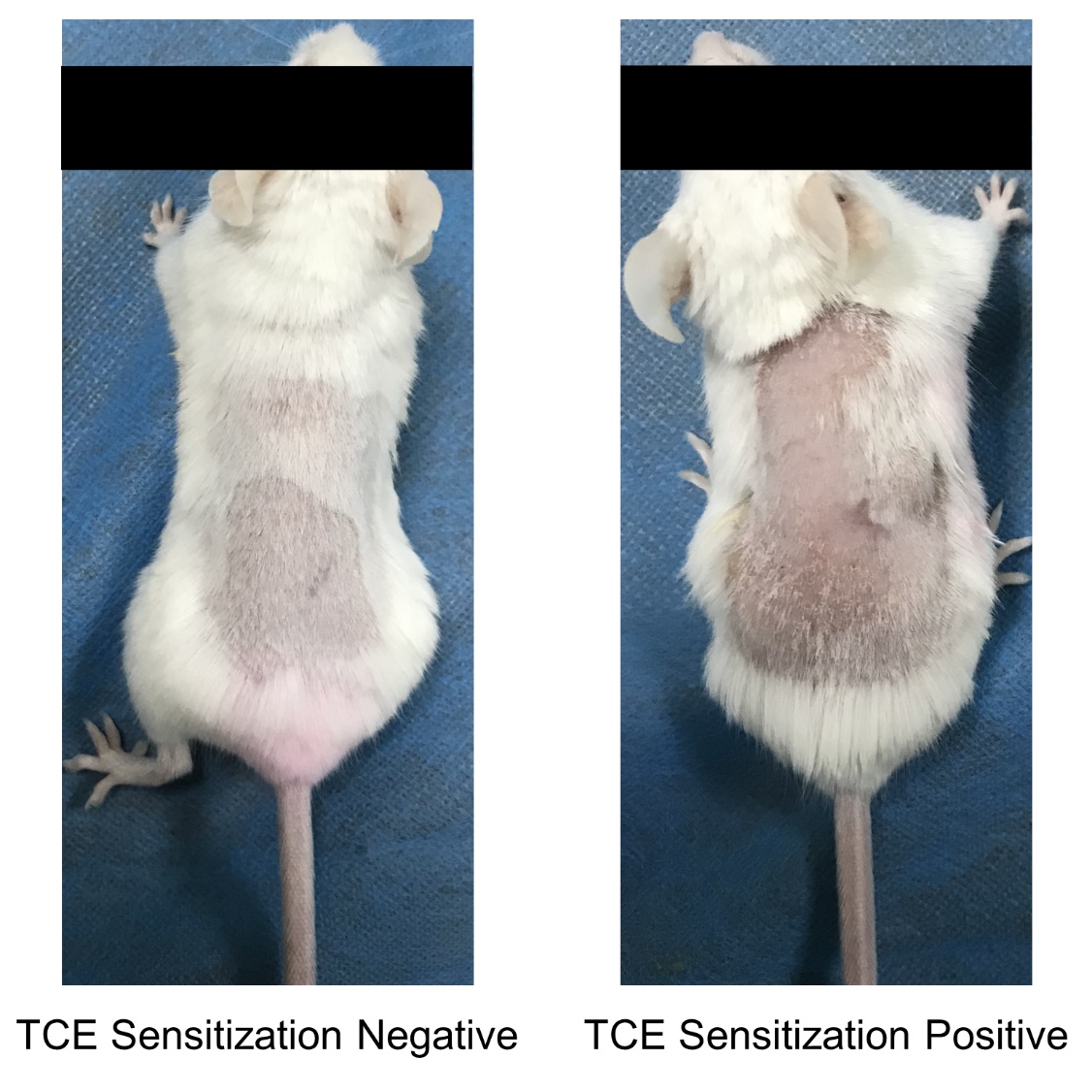


**Fig. S1. The dorsal skin reactions of TCE-treated mice.** Left: TCE sensitization negative mice; Right: TCE sensitization positive mice.

Figure S2


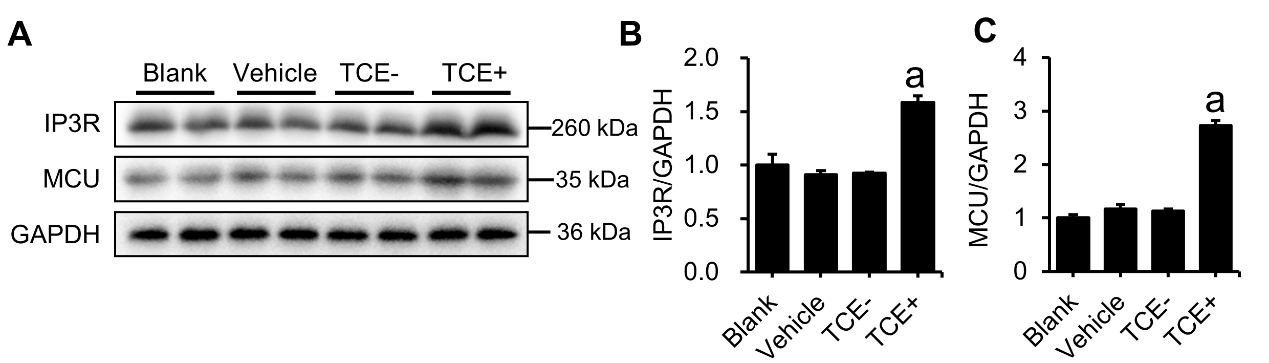


**Fig. S2. IP3R and MCU expression of TCE-sensitized mice.** (A) IP3R and MCU protein in the mouse renal cortex was detected using immunoblotting. (B and C) Quantitative analysis of IP3R and MCU protein levels. Quantitative data are shown as the mean ± SEM. (n = 4 per group). ^a^*P* < 0.05 compared with the Vehicle group, ^b^*P* < 0.05 compared with the TCE+ group

Figure S3


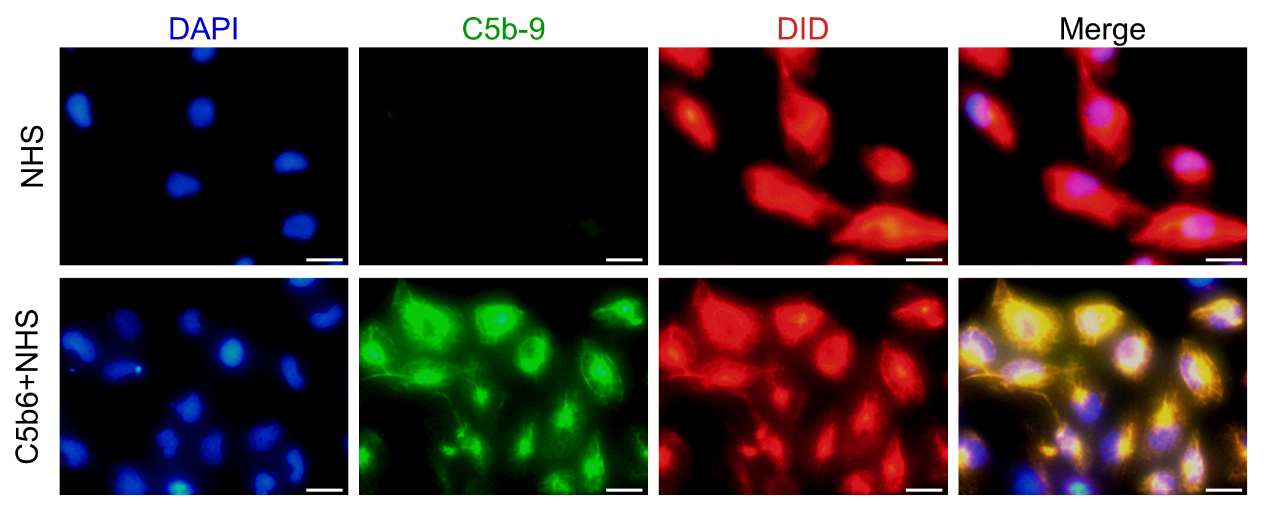


**Fig. S3. C5b-9 was assembled in HK-2 cells.** C5b-9 was measured using immunofluorescence, and the DID was used to localize the cell membrane, The scale bar represents 20 μm. (n = 6 per group).

Figure S4


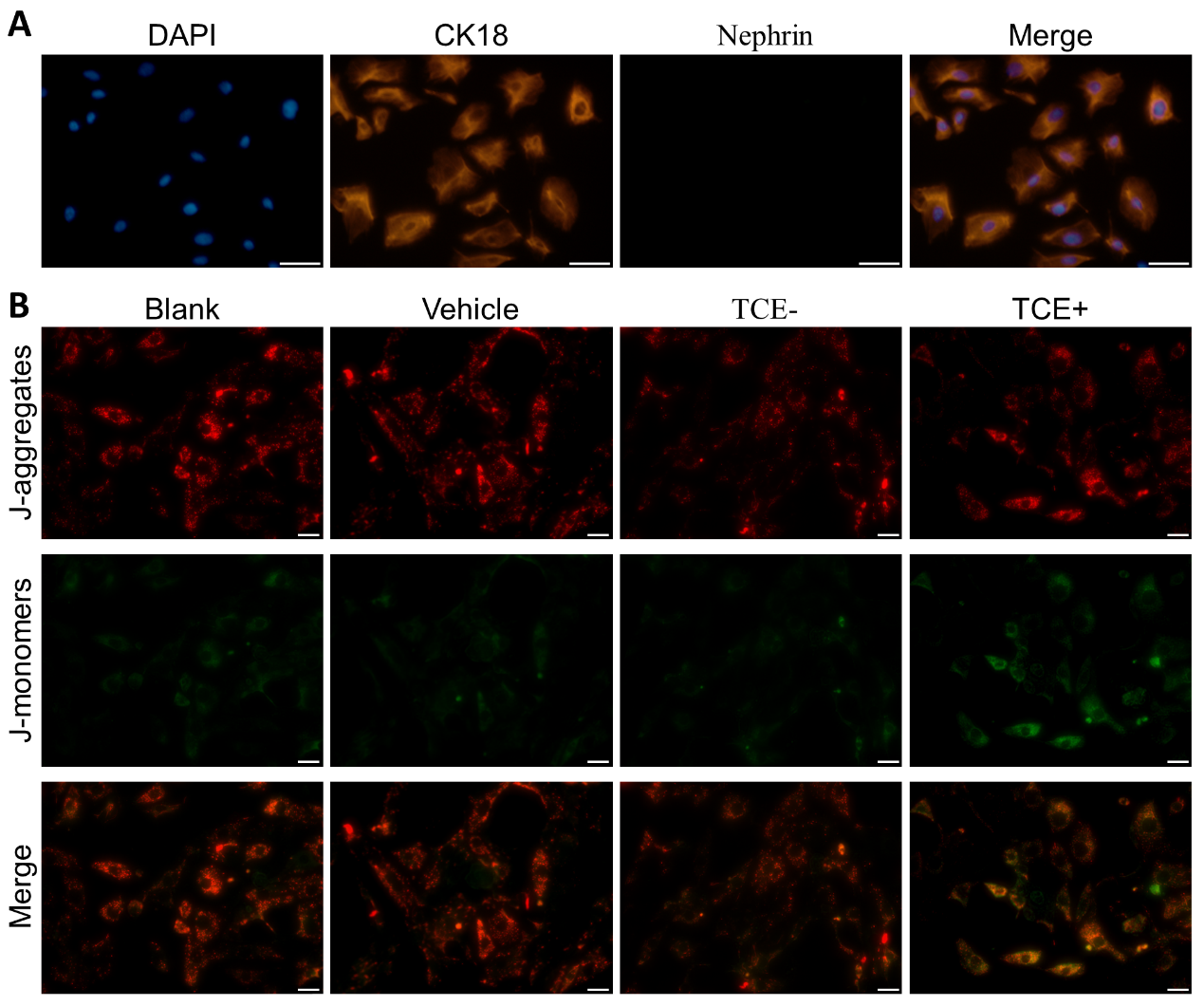


**Fig. S4. Identification and mitochondrial membrane potential of primary tubular epithelial cells.** (A) Immunofluorescence of CK18 and Nephrin was used to identify the primary renal tubular epithelial cells. (B) The mitochondrial membrane potential of TCE-sensitized mouse primary tubular epithelial cells was detected using JC-1 dye. The scale bar represents 50 μm. (n = 4 per group).

Figure S5


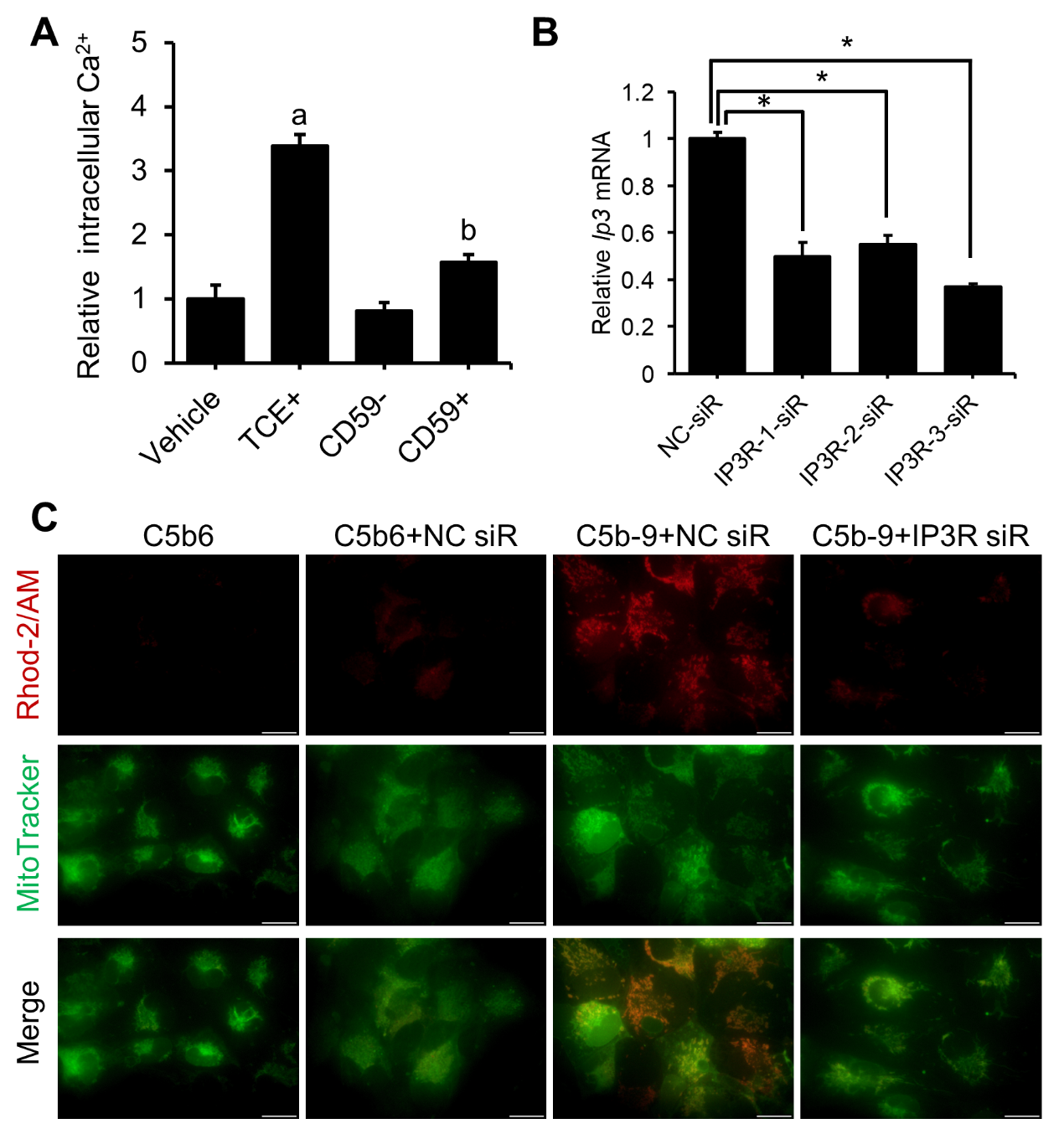


**Fig. S5. The relative Ca^2+^ levels and the efficiency of *IP3R* RNA interference.** (A) Relative cytosolic Ca^2+^ concentration of TCE-sensitized mouse primary tubular epithelial cells was detected by monitoring the fluorescence intensity at Ex/Em = 490/525 nm using a multimode plate reader (PerkinElmer). (B) The efficiency of *IP3R* RNA interference was detected using RT‒PCR. (C) The mitochondrial Ca^2+^ levels were measured using Rhod-2/AM probes and mitochondrial was marked with MitoTracker Green. The scale bar represents 20 μm. (n = 4-6 per group). ^a^*P* < 0.05 compared with the Vehicle group, ^b^*P* < 0.05 compared with the TCE+ group, **P* < 0.05.

Figure S6


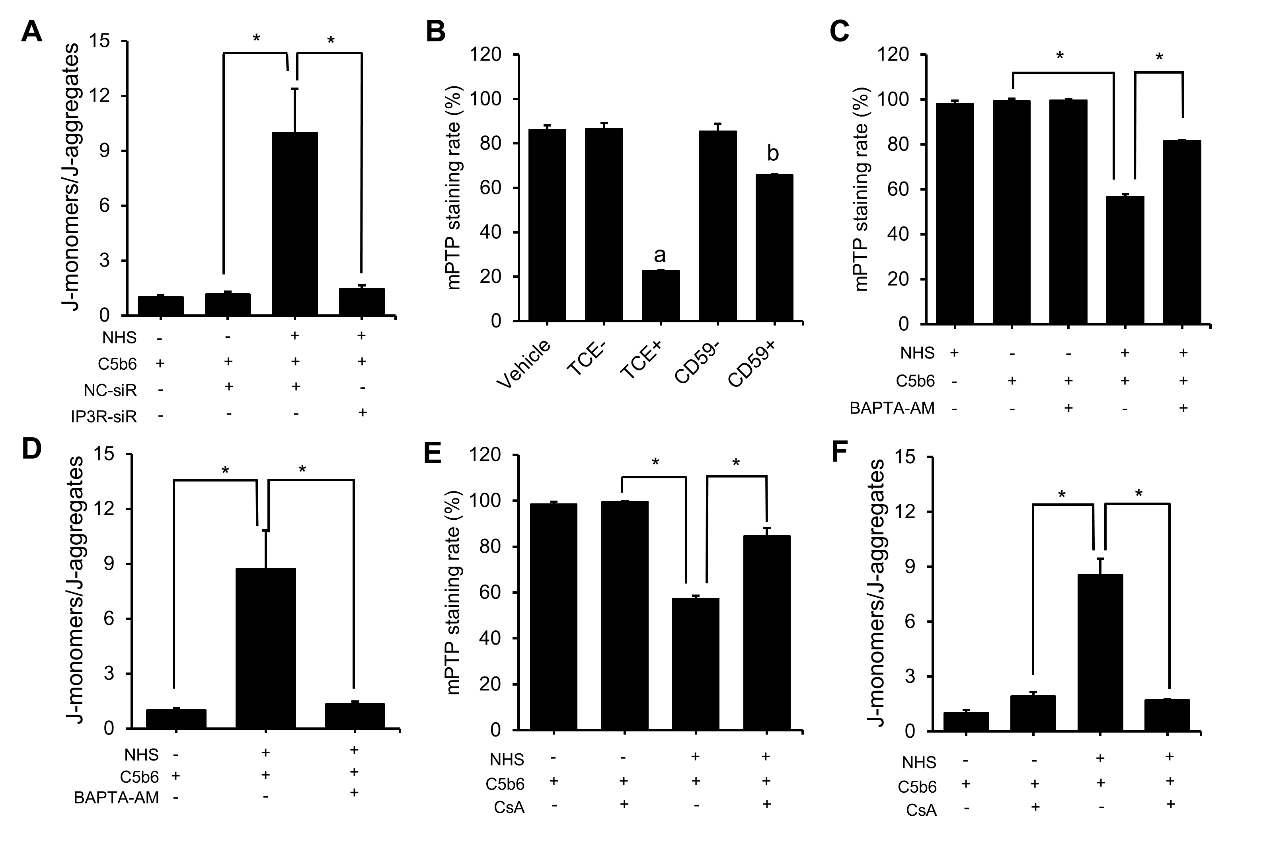


**Fig. S6. The quantified and statistically analysis of flow cytometry assays.** (A) Quantified and statistically analysis related to Fig. 3S. (B) Quantified and statistically analysis related to Fig. 5A. (C) Quantified and statistically analysis related to Fig. 5B. (D) Quantified and statistically analysis related to Fig. 5G. (E) Quantified and statistically analysis related to Fig. 6A. (F) Quantified and statistically analysis related to Fig. 6C. (n=3 per group). ^a^*P* < 0.05 compared with the Vehicle group, ^b^*P* < 0.05 compared with the TCE+ group, **P* < 0.05.
